# Molecular mechanism by which acyclic retinoid induces nuclear localization of transglutaminase 2 in human hepatocellular carcinoma cells

**DOI:** 10.1038/cddis.2015.339

**Published:** 2015-12-03

**Authors:** R Shrestha, H Tatsukawa, R Shrestha, N Ishibashi, T Matsuura, H Kagechika, S Kose, K Hitomi, N Imamoto, S Kojima

**Affiliations:** 1 Micro-Signaling Regulation Technology Unit, Division of Bio-Function Dynamics Imaging, RIKEN Center for Life Science Technologies, Wako, Saitama, Japan; 2 Graduate School of Medical & Dental Sciences, Tokyo Medical and Dental University, Bunkyo-ku, Tokyo, Japan; 3 Department of Basic Medicinal Science, Graduate School of Pharmaceutical Sciences, Nagoya University, Furo-cho, Chikusa Nagoya, Aichi, Japan; 4 Graduate School of Bioscience and Biotechnology, Department of Life Science, Tokyo Institute of Technology, Yokohama, Kanagawa, Japan; 5 Tokyo New Drug Research Laboratories, Pharmaceutical Division, KOWA Company, Ltd., Higashimurayama, Tokyo, Japan; 6 Department of Laboratory Medicine, The Jikei University School of Medicine, Nishi-shinbashi, Minato-ku, Tokyo, Japan; 7 Cellular Dynamics Laboratory, RIKEN, Wako, Saitama, Japan

## Abstract

Nuclear accumulation of transglutaminase 2 (TG2) is an important step in TG2-dependent cell death. However, the underlying molecular mechanisms for nuclear translocation of TG2 are still poorly understood. In this study, we demonstrated that acyclic retinoid (ACR) induced nuclear accumulation of TG2 in JHH-7 cells, a hepatocellular carcinoma (HCC) leading to their apoptosis. We further demonstrated molecular mechanism in nuclear-cytoplasmic trafficking of TG2 and an effect of ACR on it. We identified a novel 14-amino acid nuclear localization signal (NLS) ^466^AEKEETGMAMRIRV^479^ in the ‘C' domain and a leucine-rich nuclear export signal (NES) ^657^LHMGLHKL^664^ in the ‘D' domain that allowed TG2 to shuttle between the nuclear and cytosolic milieu. Increased nuclear import of GAPDH myc-HIS fused with the identified NLS was observed, confirming its nuclear import ability. Leptomycin B, an inhibitor of exportin-1 as well as point mutation of all leucine residues to glutamine residues in the NES of TG2 demolished its nuclear export. TG2 formed a trimeric complex with importin-*α* and importin-*β* independently from transamidase activity which strongly suggested the involvement of a NLS-based translocation of TG2 to the nucleus. ACR accelerated the formation of the trimeric complex and that may be at least in part responsible for enhanced nuclear localization of TG2 in HCC cells treated with ACR.

Transglutaminase 2 (TG2) is a multifunctional enzyme and the primary member of a family of Ca^2+^-dependent crosslinking enzymes that catalyze posttranslational modification of proteins to form N*^ε^*(*γ*-glutamyl) lysine bonds.^1^ Structurally TG2 is composed of four domains: an NH_2_-terminal *β*-sandwich domain; a catalytic core domain containing a catalytic triad for the acyl-transfer reaction (Cys277, His335 and Asp358) for acyl-transfer reaction; a *β*-barrel_1_ domain, containing GDP/GTP-interacting residues, that is involved in receptor signaling and a *β*-barrel_2_ domain.^1^ Two conformational arrangements of the four domains in TG2 have been resolved. In the GTP/GDP-bound TG2, the two COOH-terminal *β*-barrel domains are folded into the core domain rendering TG2 in a ‘closed' conformation covering the transamidation catalytic site.^2^ Similar structure was reported for ATP-bound TG2.^3^ On the contrary, the two C-terminal domains moved further apart aligning the four domains to make up an extended ‘open' conformation in a pentapeptide TG2 inhibitor, Ac-P(DON)LPF-NH_2_-bound TG2 complex^4^ and in the presence of Ca^2+^ exposing the catalytic site.^5, 6^

One of the features of the TG2 is the reciprocal regulation of the enzyme to act as a G protein in ‘closed' form or as a transamidation enzyme in ‘open' form. In as low intracellular Ca^2+^ concentrations as 10 nM, it mainly acts as a GTPase-supporting growth.^7, 8, 9^ In contrast, when cells are injured and the intracellular Ca^2+^ concentration exceeds 700–800 nM, it acts as a crosslinking enzyme suppressing cell growth and inducing apoptosis.^9, 10^ Another feature of TG2 is cellular distribution of the enzyme. While it is predominantly found in cytoplasm, it is also distributed in extracellular matrix and in various subcellular locations including plasma membrane, mitochondria, recycling endosomes and nucleus.^11^ Depending on in which milieu and what biological activity TG2 exerts, cell growth, differentiation and apoptosis are variously regulated.^8, 12, 13, 14^

Acyclic retinoid (ACR), a synthetic retinoid prevents the recurrence and development of HCC in patients after surgical removal of the primary tumors by inducing apoptosis.^15, 16^ Retinoid X receptor (RXR) *α* is highly phosphorylated and loses its activity as a transcriptional factor during carcinogenesis in HCC.^17^ ACR prevents this aberrant hyper-phosphorylation of (RXR) *α* by suppressing the Ras-extracellular signal regulated kinase (Erk) pathway, thereby restoring (RXR) *α*'s activity in response to physiological concentrations of 9-*cis* retinoic acid.^18^ A novel TG2-dependent apoptotic pathway is also involved in the apoptosis of HCC cells treated with ACR.^19^ ACR stimulates the expression, nuclear localization and crosslinking activity of TG2, resulting in crosslinking and inactivation of a transcription factor, Sp1, thereby reducing expression of epidermal growth factor receptor and inducing cell death in HCC.^19^ This novel TG2-dependent apoptotic pathway is also involved in alcoholic and nonalcoholic liver injuries.^20, 21, 22^ The nuclear abundance of TG2 appears to be particularly relevant, as this has been previously found to be associated with apoptosis in other cell types.^1, 8, 23, 24, 25, 26^ Therefore, nuclear accumulation of TG2 is of importance to regulate TG2-dependent cell death. However, its molecular mechanism is largely unknown.

Nuclear transport is a highly regulated process that dictates whether a cargo can enter into and exits from the nucleus.^27^ A variety of nuclear transport pathways occur in human cells that are mediated by different carrier molecules, each of which transports a specific range of macromolecules into or out of the nucleus.^27, 28, 29^ Human genome encodes at least 20 members of the importin-*β* family that mediates the nucleocytoplasmic transport of most proteins and RNAs.^28^ Most of importin-*β* family members, interact with nuclear localization signal (NLS) and nuclear export signal (NES) directly and translocate the containing cargoes. For importin-*β*1, in addition to the direct interaction, it utilizes importin-*α* family proteins as adaptors to bind with NLS, and mediates nuclear import of many different proteins.^28, 29^ Importin-*α* family proteins are grouped into three major subfamilies (a) importin-*α*1/Rch1/KPNA2, (b) importin-*α*3/Qip1/KPNA4, importin-*α*4/SRP1*γ*/KPNA3 and (c) importin-*α*5/NPI1/KPNA1, importin-*α*6/KPNA5 and importin-*α*7/KPNA6. The members of inter subfamilies share about 50% of sequence identity while it is at least 80% for intra subfamilies.^30^

Based on primary sequence analysis, Peng *et al.*,^31^ suggested that TG2 contains computationally predicted putative bipartite NLSs located at positions ^259^DILRR^263^ termed here as p-nls1 and ^597^PKQKRK^602^ termed here as p-nls2, as shown in Figure 1 with high homology to NLS of NS1 nonstructural protein of influenza virus.^31, 32^ They also showed that a nuclear transport factor, importin-*α*3, as a binding partner of TG2 by using yeast two-hybrid system, is involved in translocation of TG2 to the nucleus. They also confirmed the interaction of importin-*α*3 with TG2 in non-small cell lung cancer cell lines by immunoprecipitation. However, no additional mechanistic details have been identified. We previously showed that ethanol (EtOH) enhances messenger RNA expression of both TG2 and importin-*α*3 increasing nuclear TG2 in hepatic cells.^20^ TG2 also contains a computationally predicted putative leucine-rich NES at the position ^657^**L**HMG**L**HK**L**^664^, near the C terminus.^33, 34^ This is one of the generally accepted consensus NES motif (LxxxLxxL) critical for exportin-1 binding, regulating the export of proteins from the nucleus to the cytoplasm in several organisms.^33, 35, 36, 37^

A critical step controlling the TG2-dependent apoptotic pathway is nuclear localization of TG2. However, underlying molecular mechanisms of TG2 translocation from cytoplasm to nucleus still remains unclear. In this study, we found that TG2 transported into the nucleus via a novel 14-amino acid NLS located within the third domain of TG2 forming a trimeric complex ‘TG2/importin-*α*/importin-*β*', while it shuttled back to cytoplasm via a leucine-rich NES region near C terminus of TG2. The formation of the trimeric complexes was promoted by ACR, at least in part through which it enhanced the nuclear accumulation of TG2 in a HCC cell line, JHH-7 cells leading to apoptosis.

## Results

### ACR-induced accumulation of TG2 in the nucleus of HCC cells, leading to their cell death

Monitoring by time lapse of JHH-7 cells overexpressing EGFP-TG2 showed four types of cells on treatment with EtOH (control) and ACR in the presence or absence of R283, (a site-directed TG inhibitor)^13^ and Z-DON (a specific TG2 inhibitor)^38^ for 12 h, including (1) the cells that had expressed cytosolic TG2 and died, (2) the cells that had expressed cytosolic TG2 and remained alive, (3) the cells that had expressed nuclear TG2 and died and (4) the cells that had expressed nuclear TG2 and remained alive (Figure 2a). Numbers of these four types of cells in 320 *μ*m^2^ area were counted under the indicated three conditions and percentages of cells in each category against total number of cells in these four categories were calculated and plotted. The cells treated with 0.1% EtOH expressed EGFP-TG2 in the cytosol only and were in the categories 1 and 2 (25% of the cells were dead (category 1), while 75% of the cells were alive (category 2)). In contrast, in cells treated with 10 *μ*M ACR, percentage of the alive cells overexpressing cytosolic EGFP-TG2 decreased from 75 to 23% (category 2) and instead 41% of the cells were in the categories 3 and 4 (25% were dead (category 3) and 16% were alive (category 4)). They started to express nuclear TG2 4–6 h after treatment and underwent apoptosis (Figures 2a and 2b) with membrane blebbing, shrinkage 6–10 h after treatment (Figures 2b and c). These results suggested that ACR induces first nuclear accumulation of TG2 and then causes apoptosis in JHH-7 cells. To confirm the role of TG2 activity in cell death, we treated the cells with ACR in presence of 100 *μ*M R283 and 50 *μ*M Z-DON. We observed that the number of cells undergoing apoptosis after nuclear expression of TG2 decreased from 25 to 8% (category 3) in R283- and Z-DON-treated cells, while the number of living cells overexpressing the nuclear TG2 increased from 16 to 47% and 37%, respectively, in R283- and Z-DON-treated cells (Figure 2a). These data were consistent with the previous work demonstrating an importance of transamidase activity in a TG2-mediated cell death process.^19, 39^ Nuclear TG2-dependent apoptosis is usually accompanied by caspase-3-dependent cascade pathway.^19^ Actually the cells treated with 10 *μ*M ACR showed 1.3-, 1.5- and 3-folds higher levels of the activated caspase-3 than EtOH-treated control cells at 4, 8 and 12 h of treatment. Inclusion of a caspase-3 inhibitor zDEVD partially prevented ACR-induced apoptosis (Supplementary Figure 1) as reported before.^19^

### The ‘C' domain of TG2 played an important role in nuclear accumulation of TG2 and had a promising NLS sequence

**Figure 1:**
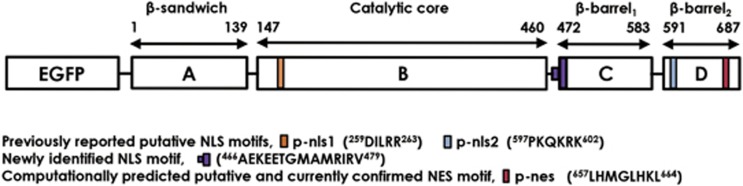
Domain structure of human TG2. Schematic diagram of EGFP-tagged human TG2 showing its four structural domains (**a**–**d**), previously reported two putative NLSs (p-nls1 and p-nls2), newly identified NLS, and computationally predicted putative and currently confirmed NES (p-nes)

We made several constructs for recombinant human TG2 domain(s)-deleted mutants tagged with EGFP at N-terminal side (Figure 3a). JHH-7 cells were transfected with these constructs whose intracellular distributions were observed in the presence and absence of ACR under a confocal microscopy. TG2 (ABC); aa1–586 that lacked ‘D' domain of TG2 thereby not containing the p-nls2 (putative NLS sequence computationally predicted in the previous paper,^31^ see Figure 1) showed nuclear localization regardless of ACR treatment (Figure 3b, row 3), while TG2 (AB); aa1–465, which lacked both ‘C' and ‘D' domains, did not localize to the nucleus even after treatment with ACR (Figure 3b, row 4). In contrast, TG2 (C); aa466–586 translocated to the nucleus regardless of ACR treatment (Figure 3b, row 5). Quantitative representations for the images in Figure 3b are shown in Supplementary Figure 2A.

Subsequently, we found that ‘AB' domain extended to the initial N-terminal 14-amino acid fragment of ‘C' domain at position ^466^AEKEETGMAMRIRV^479^ (Figure 3c, row 2), as well as this 14-amino acid fragment alone (Figure 3c, row 3) translocated to the nucleus. To verify if this novel 14-amino acid motif showed ability as NLS, chimeric recombinant human GAPDH proteins, which were fused either with SV40 NLS (PKKKRKV) or with ^466^AEKEETGMAMRIRV^479^ at N terminus and with myc-HIS at C terminus, were examined for their subcellular localization when overexpressed in JHH-7 cells (Figure 3d). The wild-type GAPDH-myc-HIS protein was found predominantly in the cytoplasm (Figure 3d, row 1); while the TG2 ‘C' domain 14 amino acid-fused GAPDH translocated in the nucleus (Figure 3d, row 3) like SV40 NLS-fused GAPDH (Figure 3d, row 2). Quantitative representations for the images in Figure 3d are shown in Supplementary Figure 2B.

### A promising role of TG2 ‘D' domain in nuclear export of TG2

TG2 (CD); aa466–687 was constructed and overexpressed in JHH-7 cells. The addition of ‘D' domain (aa587–687) to TG2 (C) sustained its accumulation in the nucleus following treatment with ACR (Figure 4a, row 2 right-hand side two panels). However, in contrast to TG2 (C) alone, this mutant did not show nuclear localization in untreated cells (Figure 4a, row 2 left-hand side two panels), suggesting that the fourth ‘D' domain of TG2 might have an important role in its nuclear export and regulation of the nuclear accumulation under ACR signal. Computer analysis for the prediction of leucine-rich NES found a putative NES at position ^657^LHMGLHKL^664^ near the C terminus domain in TG2. We treated JHH-7 cells overexpressing TG2 (ABCD) and TG2 (CD) with leptomycin B (LMB), an inhibitor of exportin-1 (Figure 4b). The inhibitor successfully induced nuclear accumulation of TG2 (ABCD) and TG2 (CD) in the absence of ACR, suggesting that D domain possess leucine-rich NES-mediating nuclear export of TG2. To confirm whether the putative NES sequence is functional, we prepared EGFP-tagged point mutation in TG2, where the three leucine residues within the putative NES sequence were all changed to glutamine (Figure 4c). Mutation of the leucine residues to glutamine residues increased nuclear accumulation of the TG2 in EtOH-treated control cells (Figure 4c, compare row 1 with row 2). Quantitative representations for the images in Figure 4 are shown in Supplementary Figures 2A and 2C.

### TG2 formed a trimeric complex with importins-*α* and *β*

To identify the co-factor(s) for nuclear import of TG2, physical interactions between TG2 and importin family proteins (importins-*α*1, -*α*3, -*α*5 and -*β*) were examined. TG2 bound with glutathione S-transferase (GST)-tagged importins -*β* (Figure 5a, lane 2), individually with importins -*α*5, -*α*1 and -*α*3 (Figure 5a, lanes 3, 5 and 7) and with the importin-*α* proteins in presence of importin-*β* (Figure 5a, lanes 4, 6 and 8). Comparing with importin-*β*, higher amounts of importin *α* subfamily proteins interacted with TG2 in the order of importins *α*3>*α*1≈*α*5 (Figure 5a), suggesting that importin-*α*3 might work as a major carrier for TG2 nuclear entry. Therefore, we used importin-*α*3 in the next experiments. In the presence of importin-*β*, increased amount of TG2 interaction was observed (compare lanes 3, 5 and 7 with 4, 6 and 8), suggesting the formation of trimeric complex of TG2/importin-*α*/importin-*β*. The interaction between TG2 and importin-*α*3/importin-*β* was not altered in the presence of Z-DON, suggesting that the interaction was independent of TG2 activity (Supplementary Figure 3). We next, explored if the 14-amino acid TG2 NLS peptide ‘AEKEETGMAMRIRV' might work as a competitive inhibitor for formation of the TG2/importin-*α*3/importin-*β* trimeric complex. We found a significant decrease in binding of TG2 with importin-*α*3 in the presence of this peptide (Figure 5b, lane 5), but not with SV40 NLS peptide (Figure 5b, lane 3) and TG2 NES peptide (Figure 5b, lane 4), thus verifying that TG2 forms TG2/importin-*α*3/importin-*β* trimeric complex, at least in part, through the newly identified NLS. We also examined the physical interactions between TG2 and exportin-1. TG2-bound, GST-tagged exportin-1 (Figure 5c, lane 2) and the binding was significantly inhibited in the presence of LMB (Figure 5c, compare lanes 2 with 7), but ACR did not interfere with TG2-exportin-1 binding (Figure 5c, compare lane 2 with lanes 4 and 5). Finally, we verified *in situ* association of TG2 with importins-*α*3, -*α*5 and -*β* using proximity ligation assay (PLA) technique. In this assay the association is shown by red dots in the cells.^40^ Each dot in the JHH-7 cell represented physical association between TG2 and importin proteins (Figure 5d, columns 2, 5 and 8).

### ACR accelerated the formation of ‘TG2/importin-*α*/importin-*β*' trimeric complex

Next we examined the effect of ACR on binding ability of TG2 with importins. We observed that incubating TG2 with ATP decreased its ability to form the trimeric complex with importin-*α*3/importin-*β* (Figure 6a, compare lane 1 with lane 4). Inclusion of EtOH restored the binding ability of TG2 and ACR further stimulated trimeric complex formation in the presence of ATP (compare lane 3 with lane 6). Effect of ACR in binding of TG2 with importin family proteins was further verified by co-immunoprecipitation of importin-*α*3 and *β*. TG2-bound importin-*α*3 and -*β* in JHH-7 cells treated either with 0.1% EtOH or 10 *μ*M ACR were co-immunoprecipitated from cell lysates using anti-TG2 antibody (CUB 7402) and determined by western blotting. We observed that binding of TG2 with importin-*α*3 and *β* in JHH-7 cells increased by 1.9- and 1.3-folds, respectively, after treatment with 10 *μ*M ACR (Figure 6b).

**Figure 2:**
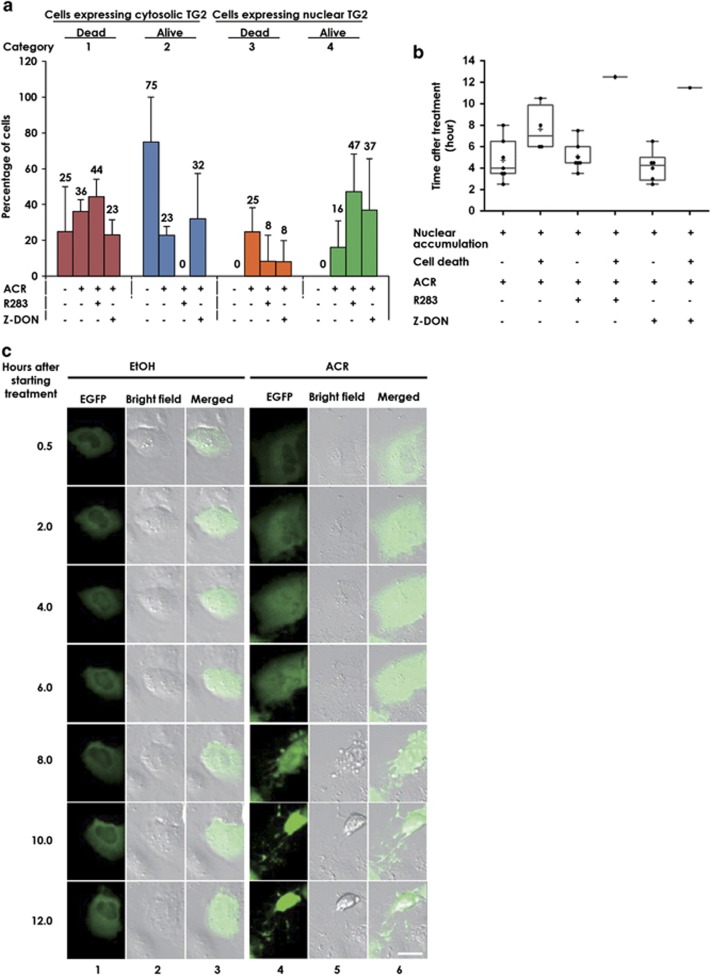
Time lapse observation of intracellular distribution of overexpressed recombinant human TG2 and cell fate in ACR-treated JHH-7 cells. JHH-7 cells were seeded at 2 × 10^5^ cells per 35 mm glass based dish coated with Type-I C collagen. The cells were transiently transfected with 0.8 *μ*g of TG2-*pEGFP-C1*. Six hours after, the cells were treated with either vehicle (0.1% EtOH) or 10 *μ*M ACR±100 *μ*M R283 or 50 *μ*M Z-DON. The transfected cells that expressed exogenously overexpressed TG2 (as judged by EGFP fluorescence intensity) at similar extent were monitored under time lapse for the next 12 h (**a**) Change in numbers of cells having the different cellular distribution of the overexpressed TG2 categorized as cytoplasmic or nuclear, and cell fate as dead or alive. Number of cells in each category out of total number of cells are expressed in percentages and plotted as bar graphs. Hundred percent represents sum of the number of cells in four categories under observation expressing exogenous EGFP-tagged TG2. Quantitated data presented as mean±S.D. of three independent experiments (*n*=3–7). (**b**) Time course changes in each category of ACR-treated cells are plotted as box and whisker diagrams showing each sample value as a dot. The lower side and upper side of boxes represent 25 and 75 percentiles in each distribution, respectively. The line and plus sign inside the box represents median and mean value, respectively. Ends of the whiskers represent either the highest or lowest sample values. (**c**) Changes in intracellular distribution of overexpressed TG2 and in the cells and their morphology monitored by the time lapse. A representative single cell treated with either 0.1% EtOH (left-hand side three columns) or 10 *μ*M ACR (right-hand side three columns) showing cell fate at the indicated time points after starting the treatment is presented. A scale bar, 20 *μ*m. A representative result from three independent experiments with similar results is presented

We also observed a time-dependent increase in *in situ* binding of TG2 and importin-*β* in ACR-treated JHH-7 cells by using PLA technique (Figure 6c). The appearance of red dots in the nucleus (shown by white arrow heads) at 5 and 7 h after ACR treatment cells signifies translocation of TG2 into the nucleus. The nuclear translocation was not observed in EtOH-treated cells. This observation is consistent with our results presented in Figure 2.

## Discussion

In this paper, we demonstrated that both, a newly identified 14-amino acid NLS located at the beginning of the ‘C' domain and ‘TG2/importin-*α*s/importin-*β*' trimeric complex formation were involved in nuclear import of TG2, while exportin-1-dependent, putative leucine-rich NES located at the ‘D' domain of TG2 was responsible for its shuttling back to the cytoplasm. We have also illustrated that ACR promoted the trimeric complex formation and induced accumulation of TG2 in the nucleus of the treated JHH-7 cells leading to their apoptosis.

Previously, we reported that crosslinking of Sp1 as a mechanism responsible for pro-apoptotic pathway by nuclear TG2 in the liver.^19, 20, 21, 22^ Here, by using time lapse monitoring of a single cell, we confirmed that ACR induces nuclear accumulation of TG2 in JHH-7 cells and leads the cell to apoptosis. Using a combination of inhibitors of TG2 activity (either R283 or Z-DON) and various TG2 mutants including the ‘AB' that has transamidase activity but deficient in nuclear accumulation (Supplementary Figure 4A), we demonstrated a link between nuclear accumulations of TG2 and exerting its transamidation activity therefore the pro-apoptotic pathway of TG2 in the liver. We also observed that apoptosis induced by ACR in cell overexpressing TG2 in cytosol cannot be prevented by a TG2 inhibitor (Figure 2a). However, co-treatment of the cells with zDEVD, a caspase-3 inhibitor and a TG2 inhibitor decreased apoptosis of both types of cells overexpressing TG2 in nucleus as well as cytosol (data not shown). This result corroborates our previous finding that both caspase-3- and TG2-mediated mechanisms for ACR-induced cell death.^19^ We observed nuclear accumulation of a TG2 mutant-lacking transamidase activity but containing both NLS and NES sequences, namely ‘CD', leading the cells to apoptosis. The rate of accumulation and extent of cell death was rather escalated by ACR (Supplementary Figure 4B). We speculate that relative better access of NLS present in C domain of TG2 (CD), which would otherwise be structurally hindered in the presence of large ‘AB' domain in the closed conformation of TG2, might be the reason for the nuclear accumulation. However, increased apoptosis by the cells expressing nuclear TG2 (CD) was unexpected. We do not know the reason, but speculate that overexpressed CD might act as a tag for endogenous TG2 to get into the nucleus given that overexpressed CD might interact with endogenous TG2. We are now testing this hypothesis.

**Figure 3:**
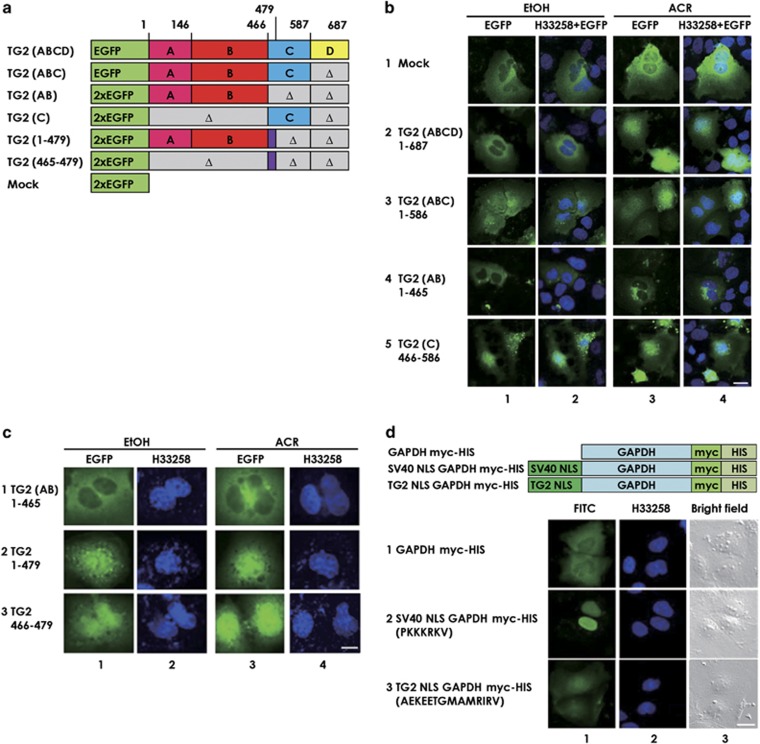
Identification of a novel NLS sequence in TG2. JHH-7 cells were seeded at 2 × 10^5^ cells per 35-mm dishes containing glass coverslips coated with Type-I C collagen, and were transiently transfected with 0.8 *μ*g of each of various EGFP-tagged TG2 and its fragments expressing vectors whose schematic structures are presented in **a**–**c**. Twenty-four hours after the transfection with the indicated vectors, the cells were treated with either 0.1% EtOH (left-hand side two columns) or 10 *μ*M ACR (right-hand side two columns) for the next 10 h. The cells were then fixed, stained with H33258 and observed under a confocal microscope. Green fluorescence from EGFP (the first and third columns) along with blue fluorescence of H33258 (the second and fourth columns) were monitored (**d**) Forty-eight hours after the transfection with the indicated GAPDH myc-HIS fused with either SV40 NLS or a novel TG2 NLS, the cells were fixed and immunostained using a FITC-tagged antibody against *myc* and co-stained with H33258. Green fluorescence from FITC (the first column) along with blue fluorescence of H33258 (the second column) was monitored under a confocal microscope. A scale bar, 20 *μ*m. A representative result from three independent experiments with similar results is presented

**Figure 4:**
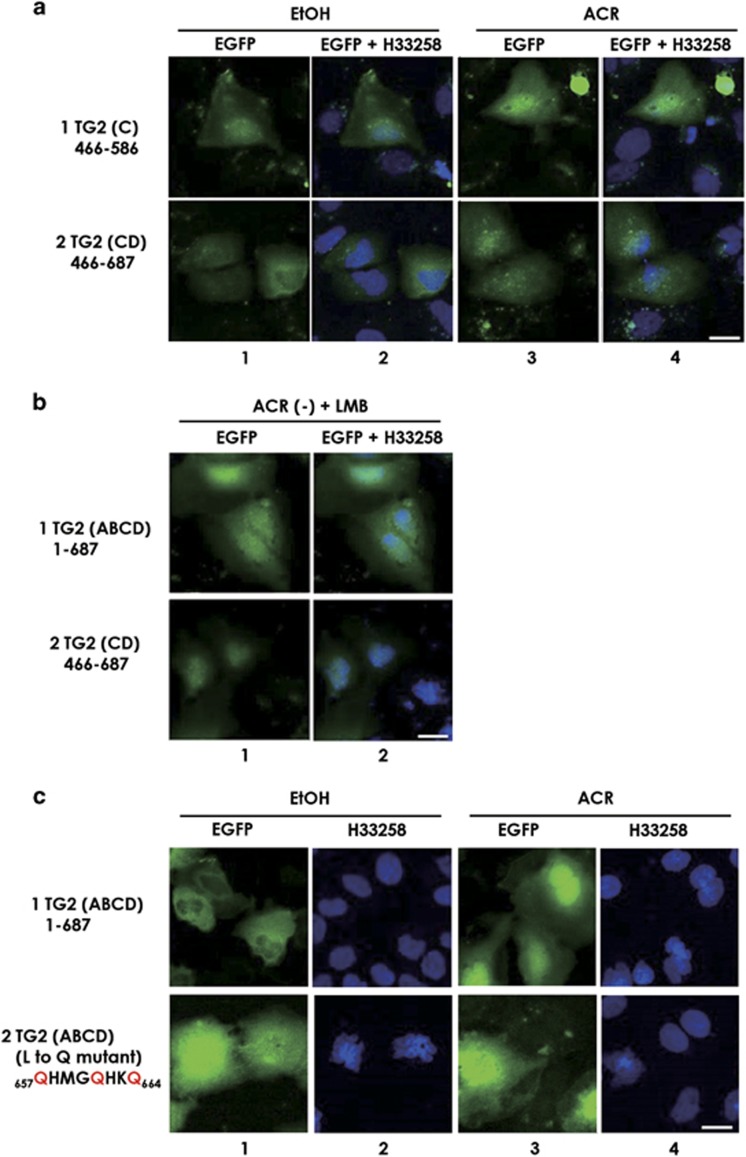
Identification of a nuclear export signal in TG2. JHH-7 cells were seeded as before and were transiently transfected with 0.8 *μ*g of the indicated EGFP-tagged, TG2 mutant-expressing vector. Twenty-four hours after the transfection with the indicated vectors, the cells were treated with 0.1% EtOH (**a** and **c**, left-hand side two columns) or 10 *μ*M ACR (**b**, left-hand side two columns; and **a** and **c**, right-hand side two columns) or 20 ng/ml LMB (**b**, right-hand side two columns) for the next 10 h. The cells were then fixed, stained with H33258 and observed under a confocal microscope. Green fluorescence derived from EGFP was monitored along with blue fluorescence from H33258. A scale bar, 20 *μ*m. Nuclear EGFP intensities for TG2 (ABCD) and TG2 (ABCD) NES mutant treated with 0.1% EtOH (**c**, left-hand side two columns) and 10 *μ*M ACR (**c**, right-hand side two columns) are presented under corresponding each panel as mean±S.D. (*n*=6). Briefly, EGFP florescence signal overlapped with H33258 florescence signal was quantitated using co-localization tool of ZEN software (Carl Zeiss, Inc., Jena, Germany) and presented as the mean EGFP intensities in the nucleus under the corresponding panels. A representative result from two independent experiments with similar results is presented

We also demonstrated that TG2 shuttled to and fro between nucleus and cytoplasm. By overexpressing TG2 fragments in JHH-7 cells, we showed that TG2 was imported into the nucleus via a newly identified 14-amino acid NLS sequence (^466^AEKEETGMAMRIRV^479^) within the ‘C' domain. The putative NLS motifs p-nls1 (aa259-263) and p-nls2 (aa597-602) are not functional in TG2 as both ‘AB' and ‘D' domain did not cause or increase the nuclear accumulation (Figures 3b, row 4 and 4a, row 2). We confirmed the nuclear localizing ability of the novel NLS by fusing it with GAPDH-tagged myc-HIS protein and verified its binding with importin-*α*3 by using the NLS peptides in competition binding assay with rhTG2. The identified novel NLS sequence is present only in TG2 among the TG family proteins but it is conserved in TG2 protein among various mammalian species (Supplementary Figure 5).

TG2 also contains a computationally predicted putative leucine-rich NES at the position ^657^**L**HMG**L**HK**L**^664^, near the C terminus.^33, 34^ We showed that the putative leucine-rich NES sequence is functional and exportin-1 dependent. Point mutation of all the leucine residues to glutamine residues in the sequence demolished the nuclear export ability of NES confirming its role in nuclear export of TG2. The NES sequence shares one of the generally accepted consensus putative NES motifs critical for exportin-1 binding, thereby being responsible for the export of proteins from the nucleus to the cytoplasm in several organisms.^35, 36, 37^ On the other hand, the NES sequence is not conserved among the TG protein family as well as among various mammalian species' TG2 protein. The sequence is unique for human and monkey's TG2 (Supplementary Figure 6).

**Figure 5:**
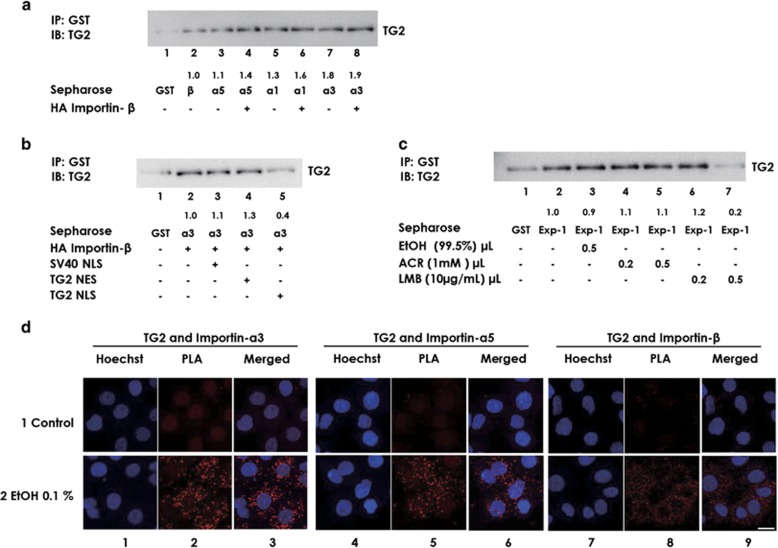
Co-factor(s) for nuclear import of TG2. Recombinant human TG2 (about 1.5 pmol) was incubated for 1 h at room temperature with glutathione sepharose 4B beads conjugated with six times molar excess of (**a**) GST (lane 1), GST-tagged importin-*β* (lane 2), -*α*5 (lane 3), -*α*1 (lane 4) and -*α*3 (lane 7) or with GST-tagged importin-*α*5, *α*1, *α*3 in presence of HA-tagged importin-*β* in (lane 4, 6 and 8 respectively). (**b**) GST (lane 1) or GST-importins-*α*3/HA-importin-*β* complex (lane 2) or in presence of peptide SV40 NLS, TG2 NES and TG2 NLS (lane 3, 4 and 5, respectively) (**c**) GST (lane 1) or GST-tagged exportin-1 (Exp-1) in the presence or absence of 0.1% EtOH, 1 mM ACR and 10 *μ*g/ml LMB as indicated. After spin-down, proteins were eluted with SDS-PAGE sample buffer and TG2 level in each pull down obtained under each condition were determined by western blotting using an antibody against TG2. A representative image, showing intensity value of each blot relative to lane 2 is presented as mean for three independent experiments (**a**) or as mean for two independent experiments (**b** and **c**). Specificity in the immunoprecipitation experiment of the TG2-exportin-1 complex was ensured in the control experiment (Supplementary Figure 7). (**d**) JHH-7 cells were seeded at 1 × 10^3^ cells per well in 96-well plate and incubated at 37 °C overnight. PLA was performed according to the manufacturer's instruction. In control (row 1), the cells were treated with media for 3 h and cells were fixed, permeabilized and no antibodies were used against TG2 and importins for PLA assay. While in others, cells were treated with 0.1% EtOH for 3 h, fixed, permeabilized and treated with combinations of mouse anti-TG2 (CUB7402) and rabbit anti-KPNA4 (left panel, row 2), mouse anti-TG2 (CUB7402) and rabbit anti-SRP-1 (central panel, row 2) or mouse anti-TG2 (CUB7402) and rabbit anti-importin-*β* (right panel, row 2). After amplification and staining with H33258, the cells were observed under a confocal microscope. Red fluorescence dots derived from amplification of detected protein interaction was monitored with blue fluorescence from H33258. A scale bar=20 *μ*m. A representative image from three independent experiments with similar results is presented

**Figure 6:**
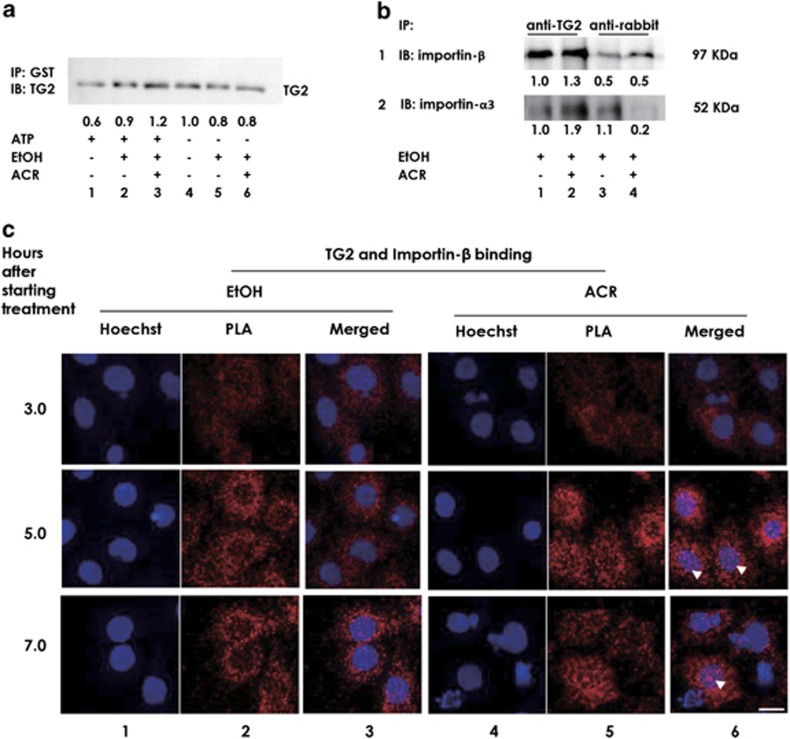
Effect of ACR in trimeric complex formation. (**a**) Recombinant human TG2 (1.5 pmol) was incubated for 1 h at room temperature with glutathione sepharose 4B beads conjugated with six times molar excess of GST-importins-*α*3/HA-tagged importin-*β* complex in the presence or absence of ATP, EtOH or ACR as indicated. After spin-down, proteins were eluted with SDS-PAGE sample buffer and TG2 levels in each co-precipitate obtained under each condition were determined by western blotting using an antibody against TG2. A representative result from two independent experiments with similar results is presented. (**b**) JHH-7 cells were seeded at 1 × 10^6^ cells per 10-cm dish overnight. The cells were then treated with 0.1% EtOH (column 1 and 3) or 10 *μ*M ACR (column 2 and 4) for next 5 h. The cells were lysed using Tris buffer (pH 7.4) with 1% Triton X-100, 0.1 mg/ml PMSF and the protease inhibitor cocktail. Importin-*β* (row 1) and importin-*α*3 (row 2) were co-immunoprecipitated using TG2 antibody (CUB7402) from samples containing equal amount of total protein determined by bicinchoninic acid (BCA) protein assay method. After precipitation, proteins were eluted with SDS-PAGE sample buffer and levels of importin-*β* and -*α*3 in each co-precipitation obtained under each condition were determined by western blotting using an antibody indicated. (**c**) JHH-7 cells were seeded at 1 × 10^3^ cells per well in 96-well plate and incubated at 37 °C overnight. The cells were then treated with 0.1% EtOH (columns 1–3) or 10 *μ*M ACR (columns 4–6) for the next 3, 5 and 7 h (rows 1–3). The cells were then fixed and PLA was performed as per manufacturer's instruction. The cells were treated with mouse anti-TG2 (CUB 7402) and rabbit anti-importin-*β*. After amplification and staining with H33258, the cells were observed under a confocal microscope. Red fluorescence dots derived from amplification of detected protein interaction were monitored with blue fluorescence from H33258. A scale bar, 20 *μ*m. A representative image from two independent experiments is presented. White arrow heads signify the nuclear TG2-importin β complex.

Previously, we reported that EtOH-treated hepatocytes showed enhanced expression of importin-*α*3, accompanied by nuclear localization of TG2.^20^ Thus, we hypothesized that importin-*α*3 may have a role in nuclear localization of TG2. Peng *et al.*^31^ reported that importin-*α*3 might be involved in the nuclear translocation of TG2 based on binding of these proteins in yeast two-hybrid analysis; however, they did not provide exact binding site for importin-*α*3. We examined the interactions between TG2 and importin-*α* proteins (Figure 5a). The current data showed that TG2 interacted with each of importin-*α*1, *α*3, *α*5 and *β* individually and in the presence of importin-*β*-forming trimeric complexes with importin-*α*1, -*α*3 and -*α*5. Importantly, the newly identified NLS sequence peptide blocked this interaction (Figure 5b). Furthermore, strikingly, the trimeric complex formation was weakened in the presence of ATP but restored and rather enhanced by ACR. The NLS sequence that we have found in TG2 has been reported to contain binding sites for ATP and GTP. TG2 in closed conformation is inactive in ATP/GTP-bound forms. We speculate that the 14-amino acid NLS sequence is available for binding when TG2 is in open conformation, and ACR might induce the conformational change in TG2 enabling it to specifically enhance nuclear accumulation of TG2 in ACR-treated HCC cells by enhancing the TG2/importin-*α*/importin-*β* trimeric complex formation. More study is needed to see the effect of ACR in the regulation of conformational change in TG2. Also, though we observed that ACR does not have effect in binding between TG2 and exportin-1, however, we cannot rule out the possibility of effect of ACR in nuclear export of TG2. We are now studying this possibility.

## Materials and Methods

### Materials

ACR (NIK-333) was provided by Kowa Company Ltd. (Tokyo, Japan). EtOH was obtained from Wako Industries (Osaka, Japan) and was used as a primary solvent. Recombinant human TG2 and Z-DON were purchased from Zedira (Darmstadt, Germany). Anti-TG2 CUB7402 was from Neomarker (Fremont, Canada). Anti-*myc*-FITC was from Invitrogen (Waltham, MA, USA). CRM1 antibody was from Thermo Scientific (Rockford, IL, USA). Anti-KPNA4, anti-SRP1 and anti-NTF97 were from Abcam (Cambridge, UK). Anti-Horseradish peroxide-conjugated goat anti-mouse IgG and anti-rabbit IgG were from Jackson ImmunoResearch Laboratories (West Grove, PA, USA). Hoechst 33258 came from Calbiochem-Novabiochem (La Jolla, CA, USA). LMB was kindly supplied by Dr. Yoshida (Chemical Genomics Research Group, RIKEN Center for Sustainable Resource Science, Wako, Saitama, Japan). A TG2 inhibitor R283 was kindly provided as a gift by Dr. Griffin (Aston University, Birmingham, UK).^13^

### Cells

A HCC cell line, JHH-7 cells were kindly supplied by Dr. Matsuura (Jikei University School of Medicine, Tokyo, Japan). The cell line was maintained in Dulbecco's Modified Eagle Medium (Wako Pure Chemical Industries, Osaka, Japan) containing 10% fetal bovine serum (Mediatech, Herndon, VA, USA), and grown at 37 °C in a humidified 5% CO_2_ incubator. For chemical treatment, the cells were cultured in serum-free media containing appropriate concentrations of EtOH or ACR.

### Plasmid and transfection

We named each of the four structural domains of TG2 as A, B, C, D domains starting from the N terminus. Expression vector for the EGFP-tagged TG2 mutants and myc-HIS-tagged GAPDH were amplified from *TG2-pSG5* vector and *GAPDH-pOTB7* vector (BC001601, Gene Engineering Division, RIKEN BioResourse Center), respectively, by PCR using synthetic oligonucleotides (Invitrogen) with appropriate restriction sites. The amplified products were ligated into *pEGFP-C1* vector (Clontech Laboratories, Inc., Mountain View, CA, USA) for EGFP-tagged TG2 mutants with EGFP at N-terminal site and *pcDNA myc-HIS 3.1* vector for myc-HIS-tagged GAPDH respectively. All the overexpressed proteins except TG2 (ABCD) showed almost similar stability (Supplementary Table 1), but this does not change the interpretation of the results presented. Cells grown for 24 h were transfected with appropriate vectors using Lipofectamine (Invitrogen) and Plus reagent (Invitrogen).

### Staining of cells

Cells grown on coverslips were fixed with 10% formalin in phosphate-buffered saline. Cells were permeabilized with 0.1% Triton X-100 in phosphate-buffered saline, and stained with antibodies as indicated in the corresponding figures. Nucleus of the cell was stained with H33258. Digital images of the cells were taken by LSM 700 laser scanning confocal microscopy (Carl Zeiss, Inc.; Jena, Germany).

### Time lapse imaging

Time lapse imagines of the treated JHH-7 cells were taken using Zeiss's LSM 700 laser scanning confocal microscope maintaining the cells at 37 °C in a humidified 5% CO_2_ incubator unit. Cells showing membrane blebbing and shrinkage, after no further shrinkage, were considered as dead cells or else alive and the time as the cell death time.

### Active caspase-3 quantitation

Quantitation of active caspase-3 was performed in duplicates using Quantikine ELISA kit (R&D systems, Inc., McKinley Place, MN, USA) as per the manufacturer's protocol. Briefly, JHH-7 cells treated with 10 *μ*M ACR or 0.1% EtOH were collected along with the detached cells using extraction buffer containing protease inhibitors at 4, 8 and 12 h of the treatment. After suitable dilution, biotin-ZVKD-fmk-labeled active caspase-3 (biotin-ZVKD-fmk is a component of Quantikine ELISA kit) in the cell lysates were measured according to the manufacturer's instructions.

### GST pull-down assay

For binding study of TG2 and importin-*α*s, 100 ng recombinant human TG2 was incubated for 1 h in Tris buffer (pH 7.6) containing 150 mM NaCl at room temperature with glutathione sepharose 4B beads (GE Healthcare, Uppsala, Sweden) conjugated with GST-tagged importin-*β*,-*α*1,-*α*3,-*α*5 or complexes of GST-tagged importin-*β* and GST-tagged importin (-*α*1,-*α*3,-*α*5), or complexes of GST importin (-*α*1,-*α*3,-*α*5) and hemagglutinin (HA)-tagged importin-*β*. For binding study of TG2 and importin-*α*s in the presence of ACR, glutathione sepharose 4B beads conjugated with GST-tagged importin-*α*3/HA-tagged importin-*β* were incubated in the presence or absence of 20 *μ*M ACR or 0.1% EtOH with 100 ng recombinant human TG2. In some samples, TG2 was pre-incubated with 500 *μ*M ATP. After spin-down, proteins were eluted with SDS-PAGE sample buffer and TG2 levels in co-precipitates under each condition were determined by western blotting using an antibody against TG2.

### Co-immunoprecipitation

Co-immunoprecipitation was performed using Dynabeads protein G as per the manufacturer's protocol. Briefly, the treated JHH-7 cells were lysed with ice-cold cell lysis buffer (Tris buffer, pH 7.4 containing 1% Triton X-100, 0.1 mg/ml PMSF and protease inhibitor cocktail). Samples were centrifuged and lysates were collected, 500 *μ*g of total protein cell lysates as determined by bicinchoninic acid protein assay were incubated with 1.5 mg Dynabeads protein G or A (Life Technologies, Oslo, Norway) pre bound with either anti-TG2 CUB7402 (0.5 *μ*g) or CRM1 antibody (0.5 *μ*g). As a control anti-mouse or anti-rabbit non-immune antibodies were also used. After binding for 15 min with the cell lysates at room temperature, the beads were washed three times with wash buffer (PBS (pH 7.4) with 0.02% Tween-20) before eluting with elution buffer (50 mM glycine (pH 2.8)) and boiled with SDS-PAGE sample buffer. Importin *α*3 and *β* levels in the precipitates under each condition were determined by western blotting using an antibody against importin-*α*3, -*β* or TG2.

### Western blotting

Western blotting was performed as before^19^ using suitable anti-TG2 CUB7402 (1 : 5000), anti-KPNA4 (1 : 2000), anti-SRP1 (1 : 1000), anti-NTF97 (1 : 300) and anti-CRM1 (1 : 1000) dilution and were probed either with horseradish peroxidase goat anti-mouse IgG (1 : 2000 dilution) or goat anti-rabbit IgG (1 : 2000 dilution). Reactants were detected with Enhanced Chemiluminescence reagents (GE Healthcare, Buckinghamshire, UK).

### Proximity ligation assay

PLA was performed using Duolink *in situ* PLA probe anti-Rabbit PLUS, anti-Mouse Minus and detection reagent red (Olink Bioscience Uppsala, Sweden) according to the manufacturer's instructions. Briefly, JHH-7 cells were treated with 10 *μ*M ACR or 0.1% EtOH. The cells were fixed after the mentioned time. The cells were then blocked and incubated with mouse anti-TG2 CUB7204 and rabbit anti-importin(s) as indicated in the corresponding figure legends. Minus probe that identifies mouse antigen and plus probe that identifies rabbit antigen were added. The minus and plus probes were ligated and the signal was amplified using ligation and development steps.^40^ The developed fluorescence was then observed under Zeiss LSM 700 laser scanning confocal microscope.

### Statistical analyses

Quantitative data are shown as mean±S.D. Statistical analyses were performed using GraphPad Prism version 6.0 for Windows (GraphPad Software, San Diego, CA, USA). *P-value<0.05 and **P-value<0.01 were considered as statistically significant.

## Abbreviations

ACR: acyclic retinoid
TG2: transglutaminase 2
HCC: hepatocellular carcinoma
NLS: nuclear localizing signal
NES: nuclear export signal
RXR: retinoid X receptor
EtOH: ethanol
LMB: leptomycin B
GST: glutathione S-transferase
