# Supplementary material for: Molecular mechanism by which acyclic retinoid induces nuclear localization of transglutaminase 2 in human hepatocellular carcinoma cells
Source: Cell Death Dis. 2015 Dec 3;6(12):e2002–. doi: 10.1038/cddis.2015.339 (PMC4720877; doi:10.1038/cddis.2015.339)
Supplement: Supplementary Information [file cddis2015339x1.docx]

**Supplementary figure 1.**

JHH-7 cells were seeded at 2 X 10^5^ cells per 35 mm glass based dish coated with Type I-C collagen. The cells were transiently transfected with 0.8 μg of TG2 (ABCD). Six hours after, the cells were treated with 10 μM ACR. The transfected cells that expressed exogenously overexpressed TG2 (as judged by EGFP fluorescence intensity) at similar extent were monitored under time lapse for the next 12 hours. Change in numbers of cells, having the different cellular distribution of the overexpressed TG2 categorized as cytoplasmic or nuclear, and cell fate as dead or alive was measured. Number of cells in each category out of total number of cells are expressed in percentages and plotted as bar graphs. A hundred % represents sum of the number of cells in four categories under observation expressing exogenous EGFP tagged TG2. Quantitated data presented as mean ± SD of three independent experiments (n = 3-7).

**Supplementary figure 2.**

JHH-7 cells were seeded at 2 X 10^5^ cells per 35 mm dish containing a glass cover-slip coated with Type-IC collagen and were transiently transfected with 0.8 μg expressing vector. A) and C) Twenty four hours after the transfection with EGFP tagged TG2 mutants, the cells were treated with the indicated treatments for next 10 hours. The cells were then fixed and stained with H33258. B) Forty eight hours after the transfection with GAPDH *myc*-HIS fused with either SV40 NLS or a novel TG2 NLS, the cells were fixed and immunostained using a FITC tagged antibody against *myc* and co-stained with H33258. Green fluorescence intensities derived from EGFP or FITC along with blue fluorescence from H33258 were monitored under confocal microscope. Transfected cells expressing nuclear TG2 mutants observed in 320 µm x 320 µm microscopic field area were classified into four categories as we indicated in figure 2 in the main text, and cell numbers in each category under each condition were counted and expressed in percentages calculated against total number of counted cells expressing exogenous TG2 mutants in the area. The percentage obtained from 4-6 microscopic fields in the same experiment is presented as mean ± SD. **p*-value <0.05, ***p*-value <0.01. A representative result from 3 independent experiments with similar results is presented.

**Supplementary figure 3.**

Recombinant human TG2 (1.5 p mole) was incubated for 1 hour at room temperature with glutathione Sepharose 4B beads conjugated with six times molar excess of GST-importins-α3/HA tagged importin-β complex in the presence or absence of ATP, EtOH, ACR or Z-DON as indicated. After spin-down, proteins were eluted with SDS-PAGE sample buffer and TG2 levels in each co-precipitate obtained under each condition were determined by western blotting using an antibody against TG2.

**Supplementary figure 4.**

JHH-7 cells were seeded at 2 X 10^5^ cells per 35 mm glass based dish coated with Type I-C collagen. The cells were transiently transfected with 0.8 μg of (A) TG2 (AB) or (B) TG2 (CD). Six hours after, the cells were treated with 0.1% EtOH or 10 μM ACR when indicated. The transfected cells that expressed exogenously overexpressed TG2 (as judged by EGFP fluorescence intensity) at similar extent were monitored under time lapse for the next 12 hours. Change in numbers of cells having the different cellular distribution of the overexpressed TG2 categorized as cytoplasmic or nuclear, and cell fate as dead or alive. Number of cells in each category out of total number of cells are expressed in percentages and plotted as bar graphs. A hundred % represents sum of the number of cells in four categories under observation expressing exogenous EGFP tagged TG2. Quantitated data presented as mean ± SD of three independent experiments (n = 3-7). (C) Time course changes in each category of (B) are plotted as Box and Whisker diagrams showing each sample value as a dot. The lower side and upper side of boxes represent 25 and 75 percentiles in each distribution, respectively. The line and plus sign in the box represents median and mean value respectively. Ends of the whiskers represent either the highest or lowest sample values.

**Supplementary figure 5.**

NCBI Align Sequences Protein BLAST was used for aligning the newly identified NLS sequence of human TG2 with A) other members of human transglutaminase family proteins B) TG2 proteins of other mammalian species. Multiple sequence alignment columns with no gaps are colored in blue or red. The red color indicates highly conserved amino acids and blue indicates less conserved ones.

**Supplementary figure 6.**

NCBI Align Sequences Protein BLAST was used for aligning the computationally predicted putative and currently confirmed NES sequence of human TG2 with A) other members of human transglutaminase family proteins B) TG2 proteins of other mammalian species. Multiple sequence alignment columns with no gaps are colored in blue or red. The red color indicates highly conserved amino acids and blue indicates less conserved ones.

**Supplementary figure 7.**

JHH-7 cells were seeded at 1 X 10^6^ cells per 10 cm dish for overnight. The cells were then treated with 0.1% EtOH (column 1, 3, and 5) or 10 µM ACR (column 2, 4, and 6) for next 5 hours. The cells were lysed using Tris buffer (pH7.4) containing 1% Triton X-100, 0.1mg/mL PMSF and the protease inhibitor cocktail. Exportin-1 was co-immunoprecipitated using CRM1 antibody from samples containing equal amount of total protein determined by bicinchoninic acid (BCA) protein assay method. After precipitation, proteins were eluted with SDS-PAGE sample buffer and TG2 level in each co-precipitation obtained under each condition was determined by western blotting using an antibody indicated.

**Supplementary table 1.**

JHH-7 cells were seeded at 1 X 10^4^ cells per well in a 96 well plate coated with Type I-C collagen. The cells were transiently transfected with 0.04 μg of A) EGFP, B) EGFP-TG2 (ABCD), C) EGFP-(ABC), D) 2x EGFP TG2 (C), E) 2x EGFP TG2 (CD), F) 2xEGFP TG2 (465-479), G) 2xEGFP TG2 (1-479). Eighteen hours after the transfection, fluorescence intensity from each the mutant was monitored till 60 hours in an interval of 6 hours using Image express. Fluorescence half-life of the overexpressed protein was calculated using formula t1/2 = [{(ln(2)*t}/ln(N_0_/N_t_)], where N_o_ is the initial fluorescence intensity and N_t_ is the fluorescence intensity at time t. N_o_ was the highest fluorescence intensity observed during the time course and N_t_ was the fluorescence intensity observed either at 6, 12, 24 or 36 hours after N_o_. An average value ± SD of three calculated t1/2 values is given.
