# Supplementary figures and images for: Molecular mechanism by which acyclic retinoid induces nuclear localization of transglutaminase 2 in human hepatocellular carcinoma cells
Source: Cell Death Dis. 2015 Dec 3;6(12):e2002–. doi: 10.1038/cddis.2015.339 (PMC4720877; doi:10.1038/cddis.2015.339)

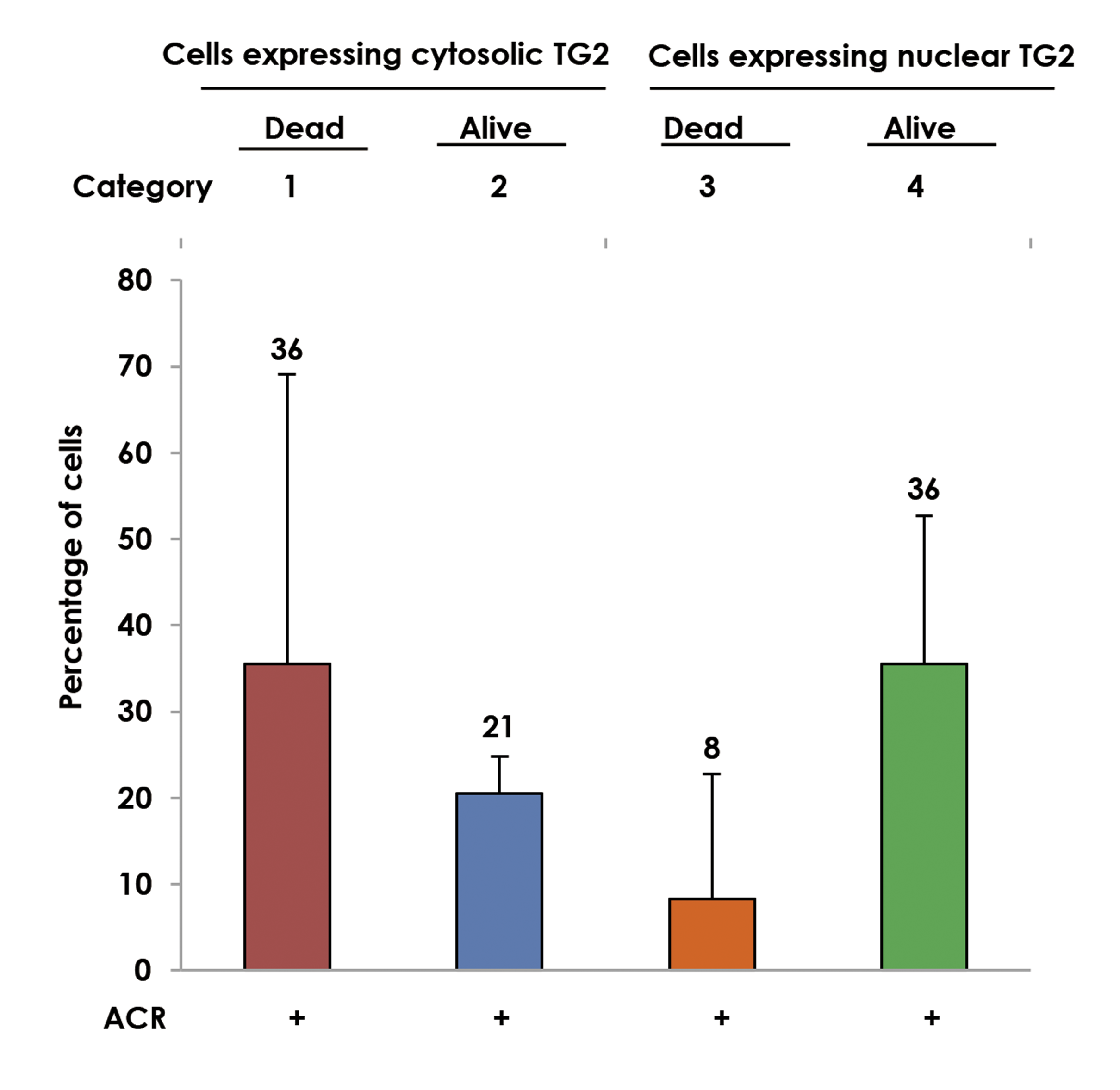

Supplement: Supplementary Figure S1 [file cddis2015339x2.tif]

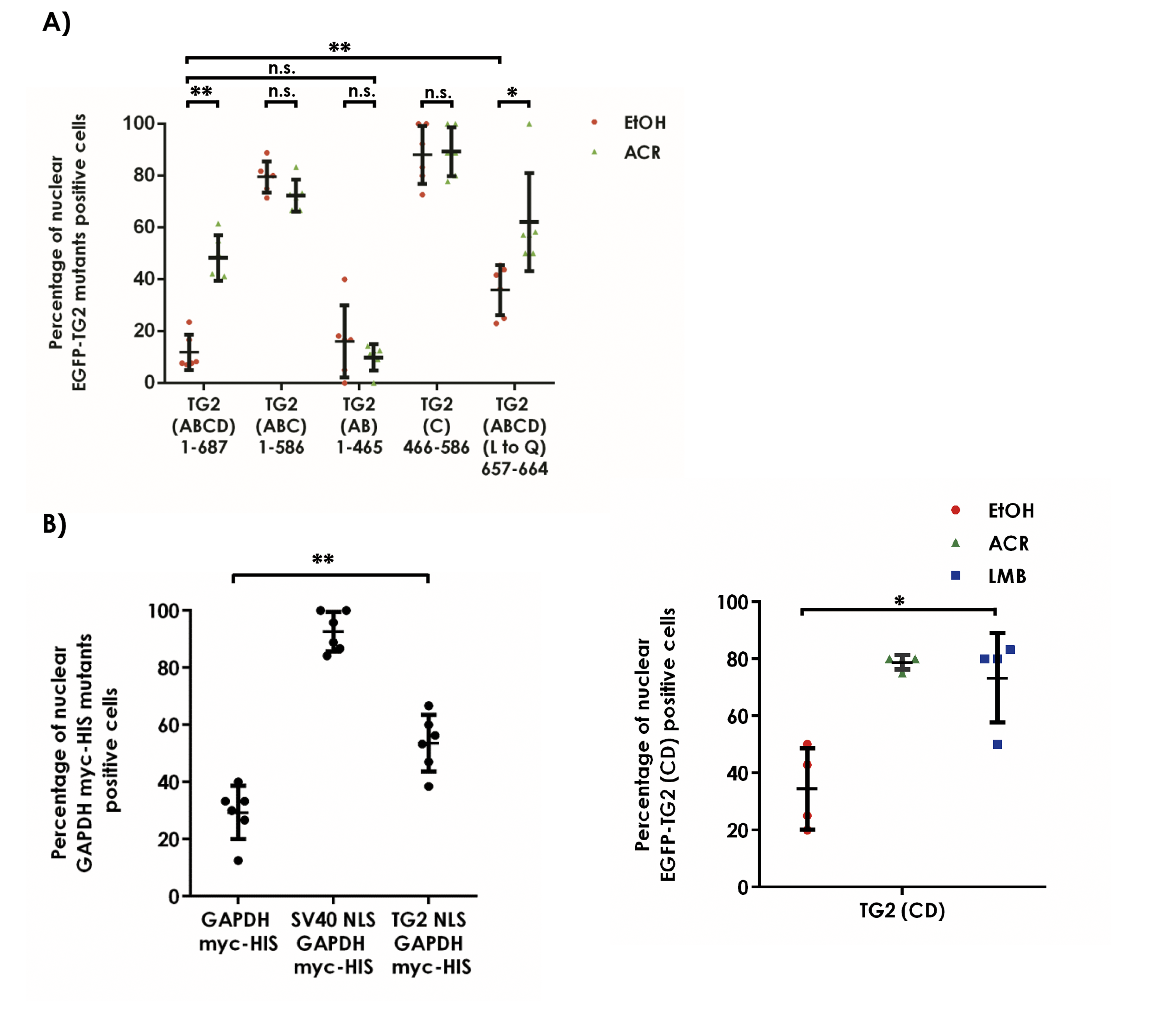

Supplement: Supplementary Figure S2 [file cddis2015339x3.tif]

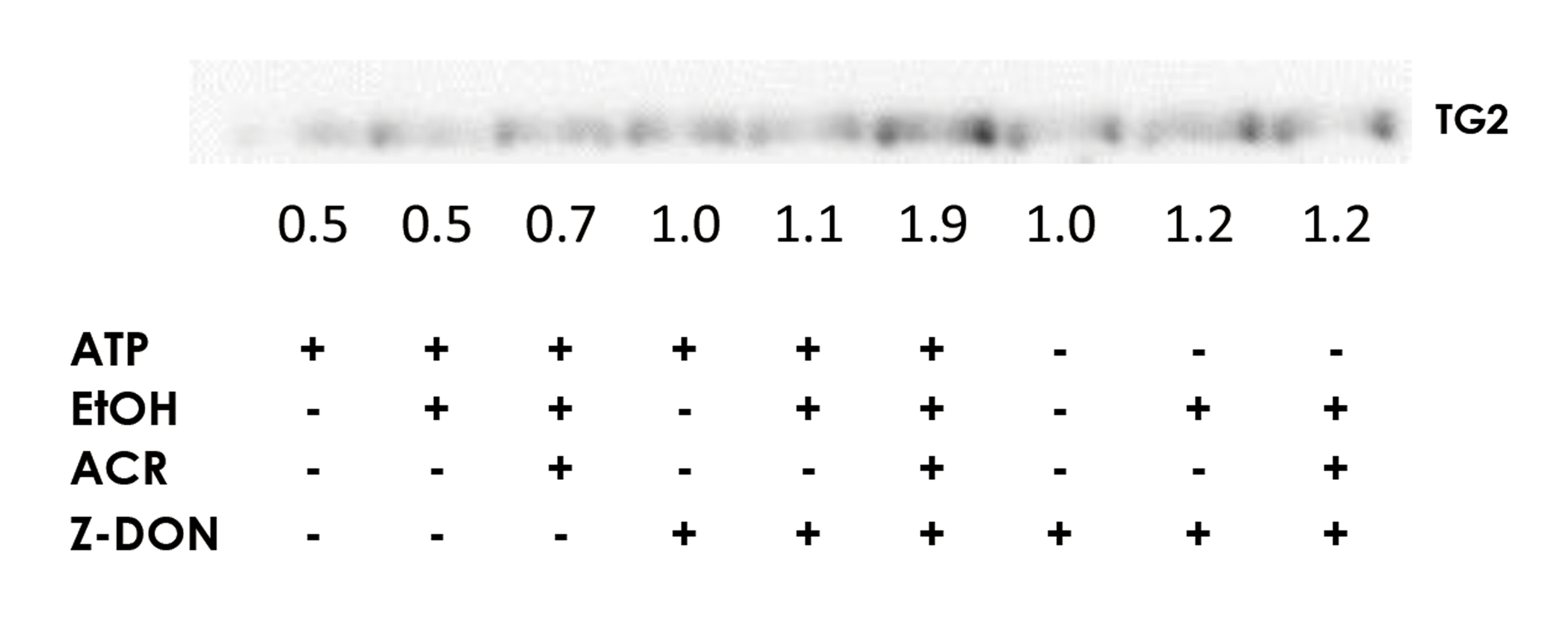

Supplement: Supplementary Figure S3 [file cddis2015339x4.tif]

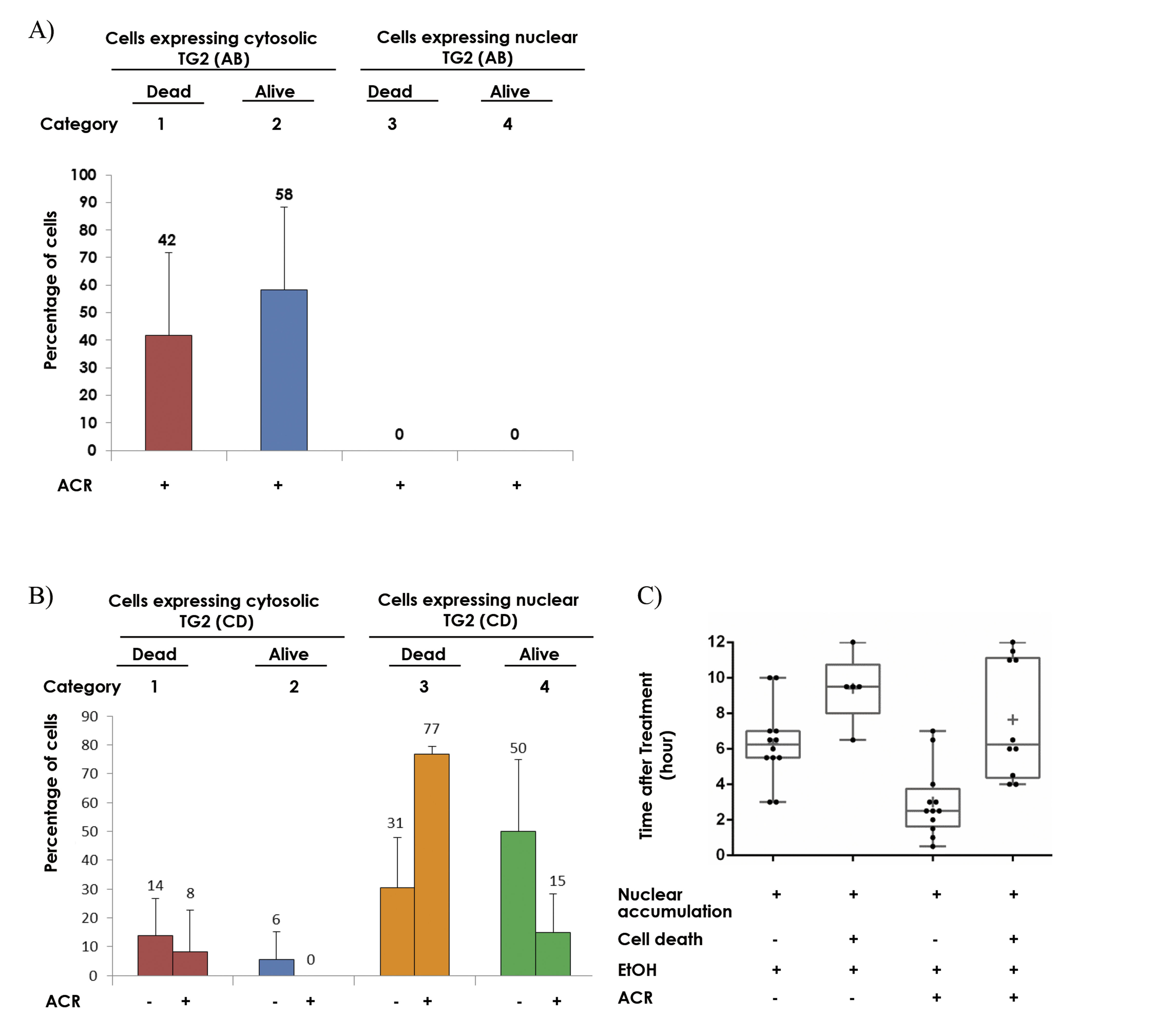

Supplement: Supplementary Figure S4 [file cddis2015339x5.tif]

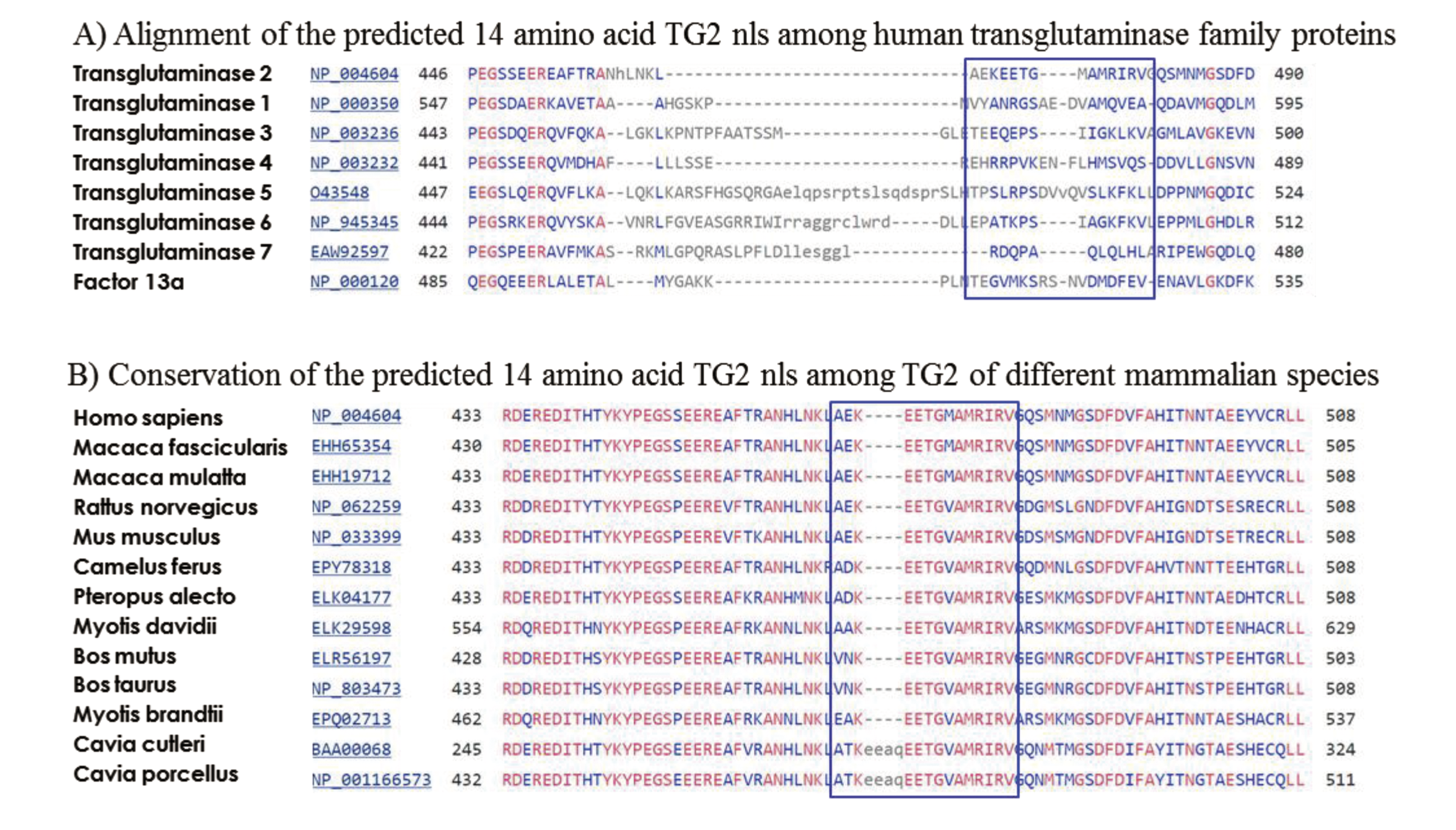

Supplement: Supplementary Figure S5 [file cddis2015339x6.tif]

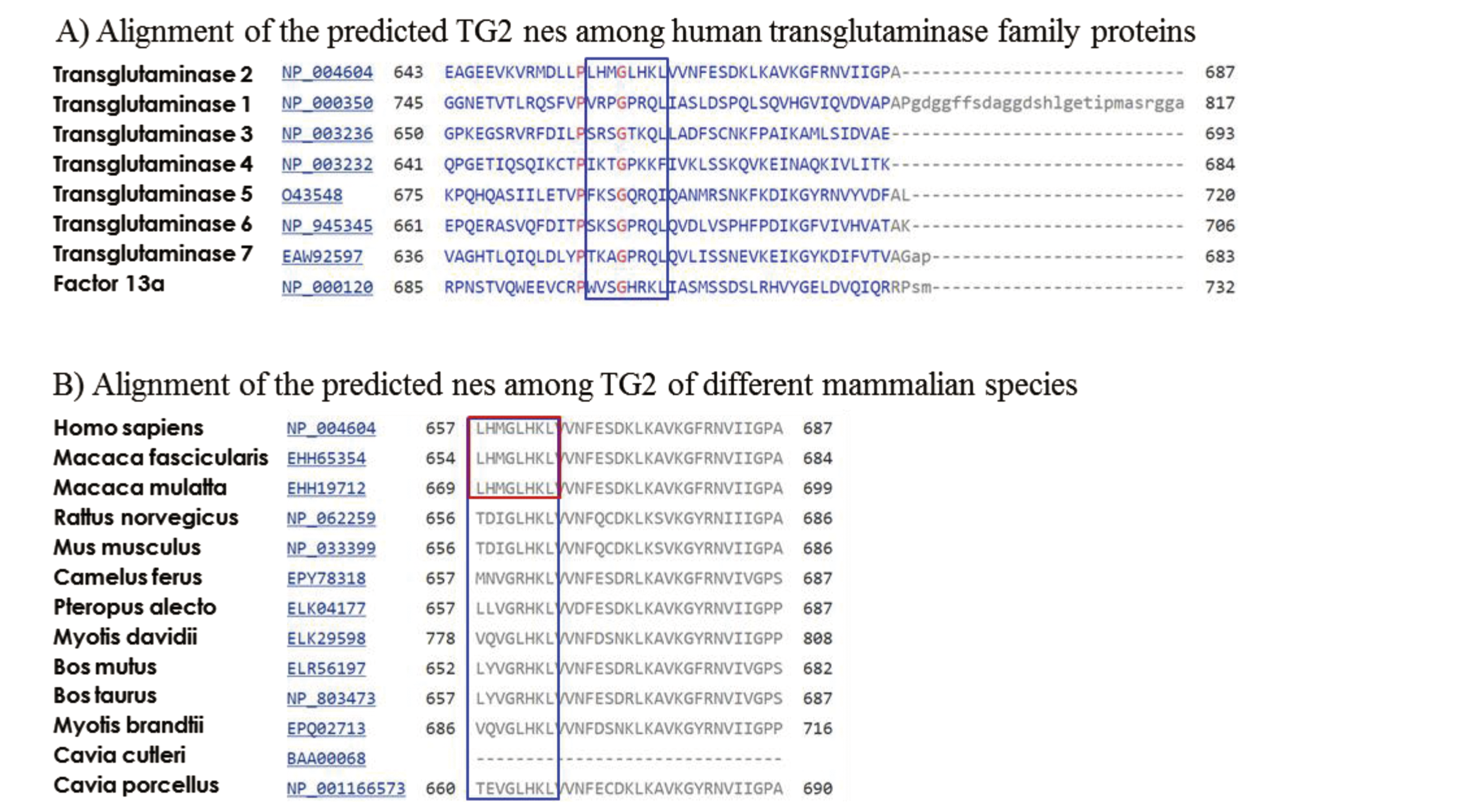

Supplement: Supplementary Figure S6 [file cddis2015339x7.tif]

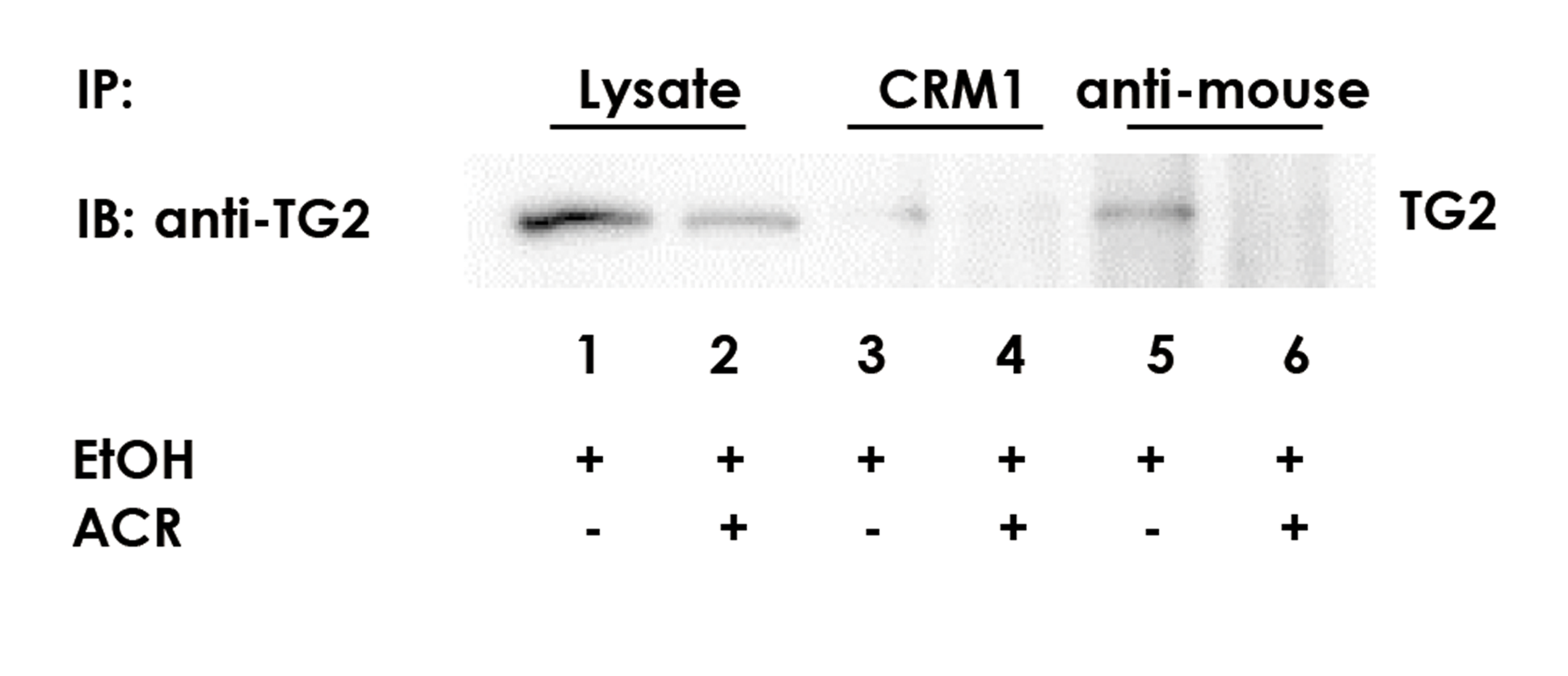

Supplement: Supplementary Figure S7 [file cddis2015339x8.tif]

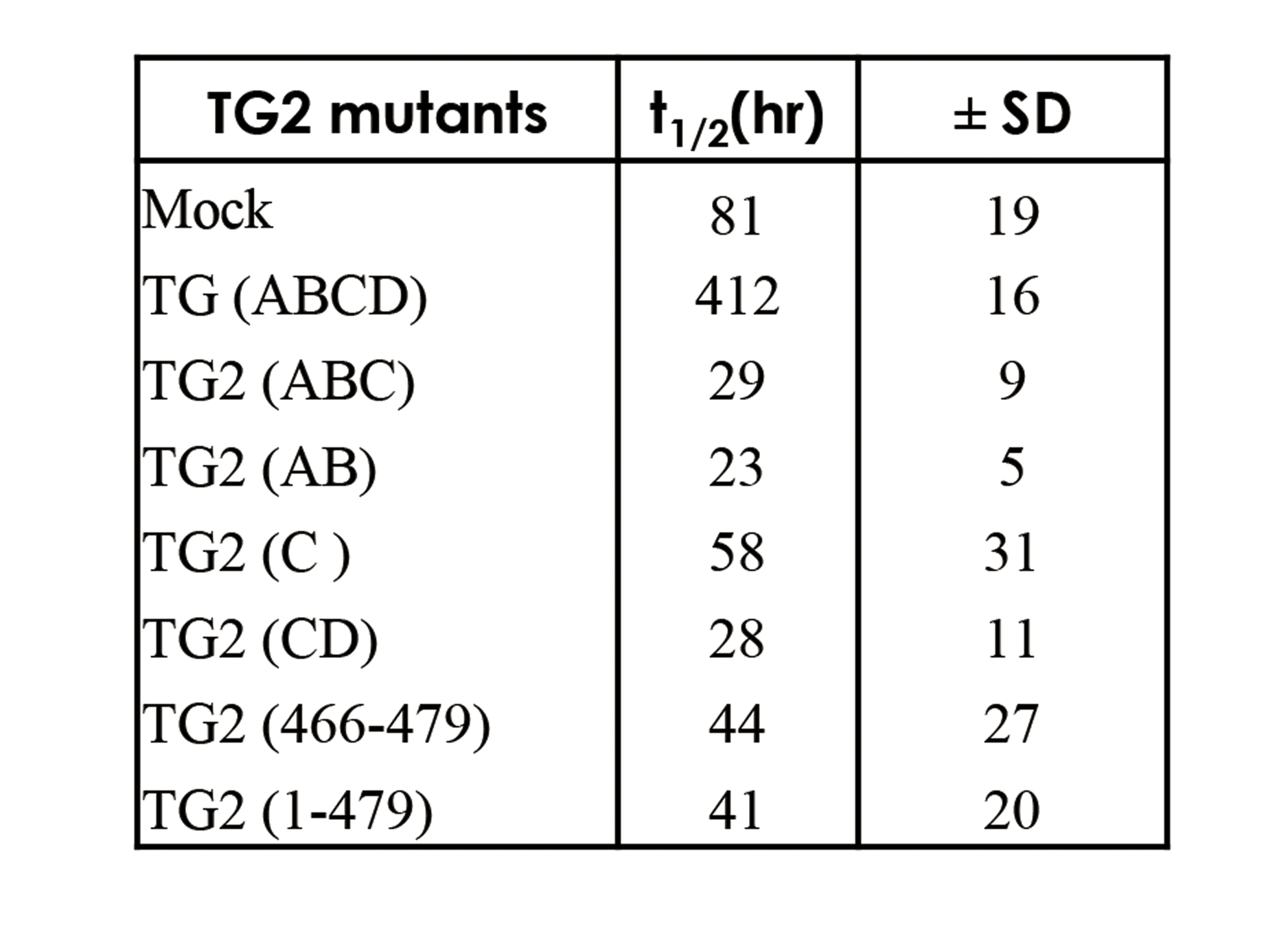

Supplement: Supplementary Table S1 [file cddis2015339x9.tif]
